# The Relationship between Formal Music Training and Conflict Control: An ERP Study

**DOI:** 10.3390/brainsci13050723

**Published:** 2023-04-26

**Authors:** Jiayi Hao, Yazhi Pang, Yong Liu, Yuanluo Jing, Jianbo Li, Ruochuan Mi, Maoping Zheng

**Affiliations:** 1 School of Psychology, Southwest University, Chongqing 400715, China; 2 Chongqing Vocational College of Culture and Arts, Chongqing 400067, China; 3 School of Music, Southwest University, Chongqing 400799, China; 4 Key Laboratory of Cognition and Personality (Ministry of Education), Southwest University, Chongqing 400715, China; 5 Chongqing Municipal Educational Examinations Authority, Chongqing 401126, China

**Keywords:** music training, conflict control, stroop task, ERP, N2, P3

## Abstract

Music training involves manifolds of sensorimotor processes that tie closely with executive functions, including conflict control. Past studies have found consistent evidence in children of the link between music learning and executive functions. However, the same relationship has not been found in adult populations, and conflict control has yet to be studied in a focused manner. Via the Stroop task and event-related potentials (ERPs), the present study examined the association between musical training and conflict control ability among Chinese college students. The findings exhibited that individuals with music training outperformed individuals without music training by demonstrating higher accuracy and faster reaction times on the Stroop task and exhibiting greater N2 and smaller P3 amplitudes compared to the control group. The results support our hypothesis that people who received music training demonstrate advantages in their capacity for conflict control. The findings also provide scope for future research.

## 1. Introduction

Cultures across the globe value music education, primarily for character-building, including sociability, dedication, and discipline, as well as for developing of talent and creativity [1,2]. Moreover, many studies have shown that music training has beneficial effects on cognitive abilities [3,4,5,6]. Sensorimotor and cognitive abilities can be intervened with and improved with training, which has a positive overall effect on cognitive abilities, according to research [7,8,9]. Thus, one prominent explanation for the beneficial effects of music training on cognitive abilities is that musicians are constantly training their cognitive capabilities. During practices and performances, musicians need to monitor their sensorimotor nuances, and quickly adjust the next performance according to the feedback of the auditory and proprioceptive systems, while also inhibiting the distracting interference of novel internal and external information [6,10,11]. 

### 1.1. Conflict Control and Its Neural Markers

Conflict control refers to the brain’s cognitive ability to monitor and resolve conflicting information in favor of goal-achieving behavior [12,13,14,15]. It is a key component in various cognitive processes since humans often perform tasks that require the simultaneous processing of conflicting information (e.g., someone refraining from speaking in a native language). When conflicts are not efficiently detected and resolved, individuals are hindered in their attention, self-regulation, and pursuing their goals [16,17,18,19]. Moreover, a past study found that patients who have experienced traumatic brain injury would repeatedly make the same mistakes, even when correct feedback was given, and studies have concluded the cause to be the impairment of conflict control ability [20,21]. 

The Stroop Color and Word Test (SCWT) is an extensively used tool in psychology for gauging an individual’s ability to block out distractions arising from conflicting information presented as a stimulus. It is commonly employed in both clinical and experimental psychology [22]. What participants would encounter during a classical SCWT are congruent and incongruent stimuli, which would be the name of a color (e.g., yellow) shown in the corresponding color (yellow) or the name of a color (e.g., green) shown in a mismatched color (e.g., red). Participants would be instructed to respond to only the color of the word and ignore the meaning of the word. The response accuracy, number of errors, response time, and completion time are often recorded for later analysis [23,24]. Although the Stroop task is primarily used to assess conflict control abilities, it also indicates other cognitive abilities, including cognitive flexibility, information processing speed, and selective attention [25,26]. Past studies have concluded that congruent stimuli require fewer cognitive demands than incongruent stimuli, while less reaction time and fewer errors are associated with better cognitive capabilities [12,27,28].

N2 (negative 2) and P3 (positive 3) are two hallmark event-related potentials (ERPs) components that are related to conflict control [29]. ERPs are tiny voltages generated in the brain when encountered with a stimulus or event and can be non-invasively measured by electroencephalography [30,31,32]. The components are usually assessed by amplitude, which refers to the difference between the baseline amplitudes before stimulus onset and the peak waveform in its time window [33,34]. The N2 is a negatively deflected and stimulus-responsive ERP that peaks around 200–350 ms after the stimulus onset [35]. The N2 component exhibited bigger amplitudes when more conflict control resources are recruited for monitoring and detecting conflicts [12,36]. Folstein and Van concluded that the N2 component holds subconstructs of the frontocentral N2 region of the brain, which refers to the activities detected that are located behind the forehead, and the posterior N2 region of the brain, which are the brain activities shown in the rear parts of the scalp [37,38,39,40]. The frontocentral N2 and posterior N2 relate to cognitive control and visual attention, respectively [35,41,42,43]. Past studies have found that reduced N2 amplitudes are associated with less ability to detect and monitor conflict during the information process [44,45,46]. The P3 component spikes positively at a typical time window of 300–500 ms after the stimulus onset and is associated with conflict control during attention and working memory processing [33,34,41,42,47,48,49]. Past studies on conflict control had found that when participants were asked to suppress a motor response, higher P3 amplitudes were exhibited, which indicated that higher cognitive efforts were required to withhold and inhibit a response [50,51].

### 1.2. Music Training and Executive Function

Executive function defines a set of cognitive abilities that control and operate various complex cognitive processes to achieve a specific goal flexibly [52]. Key cognitive abilities include working memory, conflict control, attention, problem-solving, and planning [53]. Past longitudinal studies have found consistent results indicating that musicians outperform controls on various paradigms that assess cognitive abilities. Many researchers concluded that the outperformance was related to superior executive function [54]. Hennessy and colleagues found that among a music training group, an athletic training group, and no training group, children in the music training group exhibited the best performance in the Stroop task and the Flanker task. In the Flanker task, in each trial, children were shown five arrows pointing either to the left, to the right, or pointing up. The children were told to press either a left or a right key depending on the arrow at the center of the screen while ignoring the other four arrows.

The study found that children who have received three to four years of music training outperformed children who did not receive such training and those who had trained athletically for three to four years. Furthermore, more years of music training resulted in greater accuracy and a shorter reaction time in both the Stroop and Flanker tasks [55]. In Moreno and colleagues’ longitudinal study among preschool children, they compared the performance of a go/no-go task, a paradigm used to assess inhibition ability, between a music training and a visual arts training group. Two computerized training programs generated by the researcher were provided to the participants. The only difference between them was the training’s content. The two programs were equal in terms of graphic settings, duration, break time, teaching staff, and learning objectives. The visual art training included the development of visuospatial skills relating to ideas such as shape, color, line, dimension, and perspective. The music training contained content on rhythm, pitch, melody, voice, and basic musical concepts. For five days a week, there were two one-hour training sessions per day. The study found that after a short-term training of only 4 weeks, children in the music training group outperformed not only the visual arts group in the post-test of the go/no-go task but also their own pretest, while this pattern was not present in the visual arts group [56]. Moreover, in another study, healthy elders aged 60 to 85 years were randomly assigned to two groups, one group listening to music for 16 weeks and the other receiving piano courses for 16 weeks. The piano course covered topics such as scales, finger dexterity practices, fundamental music theory, and well-known piano literature. Participants had to practice for 30 min every day in addition to the 45 min of weekly lessons. The group also met for a 45-min music appreciation session once a week and had to spend 30 min each day listening to CDs. The music listening group listened to both world music and classical vernacular music. The result shows that older adults who received piano courses performed better on the Stroop task than those who simply received listening to music [57]. Another study exploring the effects of music training on older adults found similar results. Participants were randomly divided into piano training and physical exercise training for 4 months. After controlling for age and education, it was found that elders who received piano training for one hour every week showed fewer interference effects in the Stroop task than those who received a physical exercise of their choice [58]. These results suggest that music training is beneficial for improving cognitive control in both children and the elderly [59].

However, while past studies have provided supporting evidence for the advantageous effects of music training on various cognitive abilities, prior studies on conflict control abilities in particular have found mixed results. A study compared the performances of the Stroop task between the music training group and the control group and found that the N2 amplitude of the former group was significantly greater than that of the controls. Based on the increased N2, the study concluded that the music training group had a stronger conflict-monitoring ability [60]. Similarly, another study has shown that professional musicians exhibited a smaller Stroop effect on the Word–Color Stroop task than amateur music lovers, which indicated that they were more capable of resolving conflicting information [61]. However, some studies have revealed a lack of a significant relationship between music training and enhanced conflict control ability. A previous study compared the performance of a Stroop task between children who have attended instrument lessons for a minimum of two years and adults who have obtained or are pursuing a music performance degree with untrained children and adults. The study found that the musicians did not exhibit higher accuracy and lower reaction time than the non-musician children and adults [62]. Slevc and colleagues [10] investigated the link between music training and three components of executive function, including conflict control, memory updating, and switching. The study found that higher musical skills were positively related to memory updating, but unrelated to conflict control and switching. Therefore, whether music training is related to conflict control ability has not been fully confirmed.

### 1.3. The Present Study

Past studies have demonstrated the advantageous effects of music training on various cognitive capabilities including verbal intelligence, working memory strength, selective attention, and information processing speed in children [3,4,54,63,64]. However, research on this effect on adult populations has been largely overlooked, while its influence on conflict control ability has yet to reach a convergent answer in the existing literature. Considering the scarcity of research on the effects of music training in adult populations, and the lack of consensus on the effects on conflict control, the current study set out to investigate the differences in the performance of a Stroop task in adults with formal music training and adults without. The study aimed at exploring the neural correlates of the relationship between conflict control in a focal manner, through the use of electroencephalogram technology, providing new perspectives for the research in related fields. We hold the hypothesis that (1) music training experience is related to enhanced conflict control ability, (2) which will be demonstrated by a topped performance in the Stroop task through faster and more accurate responses, (3) and differences in the N2 and P3 amplitudes recorded during the task.

## 2. Methods

### 2.1. Participants

Participants of the current study were recruited from Chong Qing Vocational College of Culture and Arts, Chongqing, China. A total of 51 college students were included in the present study. Twenty-five participants who major in Music (including vocal, piano, violin, Chinese zither, trumpet, tube, and clarinet) were categorized as the music training group (14 females, Mage = 19.40, SD = 1.32; training years = 5.84 years, SD = 1.38;); and the other 26 participants who majored in other studies and have not received any formal music training prior to the experiment were categorized as the control group (14 females, Mage = 20.15, SD = 0.92). Each participant reported having normal or corrected-to-normal vision, was right-handed, and had no prior history of neurological or psychiatric disorders. Prior to their involvement in the study, written consent was obtained. The study was approved by the Ethics Committee of Southwest University (No. H22002).

### 2.2. The Raven Advanced Progressive Matrices

The Raven Advanced Progressive Matrices (RAPM) is a test widely used to measure higher-order general cognitive ability and to estimate general intelligence [65]. It contains 12 pattern-matching problems that become progressively more difficult. Each problem contains a diagrammatic puzzle that is missing one piece, and test takers must choose the correct missing piece from a list of options. The test is scored by summing up the number of problems solved correctly, whereby higher scores indicate better performance on the test. The test holds a Cronbach alpha of 0.79 to 0.80 [66].

### 2.3. The Stroop Task

**Figure 1:**
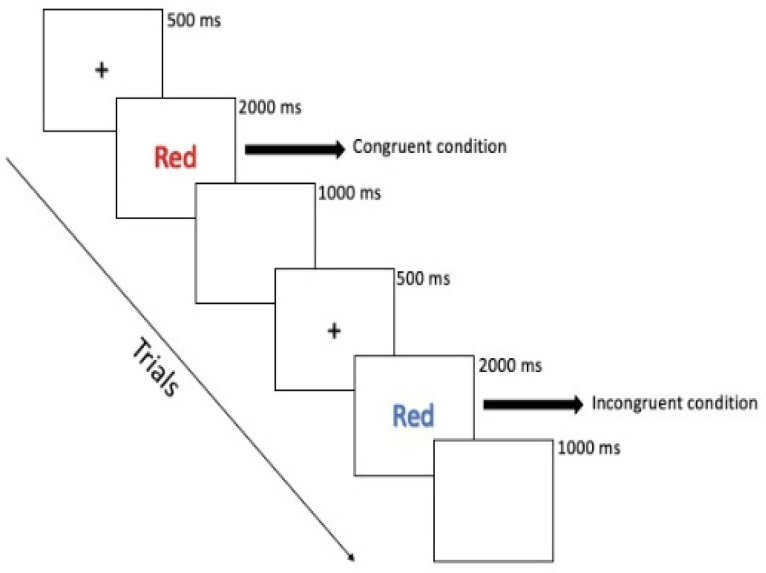
The Stroop task trials. In a congruent trial, the word “red” is shown in red ink. In an incongruent trial, the word “red” is shown in blue ink.

Four words—red, blue, green, and yellow—shown in either red, blue, green, or yellow colors made up the stimuli of the Stroop task used in the present research. In the present Stroop task, there are two different kinds of trials. When a trial is congruent, the stimulus word is displayed in the color that corresponds to it (for example, when “blue” is shown in blue ink); however, when a trial is incongruent, the stimulus word’s color does not correspond to its meaning (for example, when “blue” is shown in green ink). Participants were instructed to respond to the color of the word as fast as possible and ignore the meaning of the word. As shown in Figure 1, for each trial, a fixation cross “+” would first appear at the center of the screen for 500 ms, after which the stimulus was presented for 2000 ms or until a response was given, followed by a blank screen of 1000 ms, completing a single trial. The participants were instructed to press the “D” key on the keyboard for red, the “F” key for green, the “J” key for blue, and the “K” key for yellow. A correct response to the color of the word counts for an accurate response. The task consisted of one practice block of 20 trials and two experimental blocks of 160 trials. Only data from the experimental block were used for later analysis. Participants were allowed to take a 1 min break between each block if they wished to take it.

### 2.4. Statistical Analysis

Data analysis was conducted using SPSS 22.0 (IBM, New York, NY, USA), and the Greenhouse–Geisser method was used to adjust *p*-values for sphericity. Multiple comparisons were also made using post-hoc *t*-tests with Bonferroni correction. An independent-sample *t*-test was conducted to compare the mean RAPM scores of the music training group and the control group.

### 2.5. Behavioral Analysis

The behavioral data of the study are the participant’s task accuracy and reaction time. The task accuracy in the Stroop task was calculated by summing up the number of trials responded to correctly by the participant and dividing that number by the total number of trials. The mean accuracy of the group was later calculated and used in the analysis. Two repeated-measure ANOVAs [2 (group: music training and control group) × 2 (condition: congruence and incongruence)] were conducted on the accuracy (ACC) and reaction time (RT), with the group as a between-subjects factor and the condition as a within-subjects factor.

### 2.6. Eeg Recording and Analysis

Brain electrical activities were recorded through an elastic cap (Neuroscan, Charlotte, NC, USA) of 32 scalp sites mounted with tin electrodes, with one reference electrode placed on the frontal central aspects (REF) (reference electrode) and a ground electrode on the medial frontal aspect (GRD). The vertical electrooculogram (EOG) (the measured eye movements) was recorded with an electrode placed on the infraorbital area of the left eye. All inter-electrode impedance was maintained below 5 kΩ during the conductive gel application and throughout the recording. 

MATLAB R2014a (MathWorks, Natick, MA, USA) and the EEGLAB toolbox 14.1.1b [67] were used for the data processing. Grand average ERPs for both the congruent and the incongruent trials were made, while only trials with the correct responses were included in the final averages. To begin, the data were reduced from 1000 to 256 Hz. A high-pass filter of 0.1 Hz and a low-pass filter of 45 Hz were applied. The left and the right mastoids were taken as reference sites. To analyze the data accurately, it was broken into small segments from 200 ms before the stimulus onset until 1000 ms after and were baseline-corrected to the pre-stimulus interval. Trials with artifacts from the electrooculograms (EOG) (e.g., ocular movements and blinks), the amplifier, and the electromyographic activities, as well as peak-to-peak deflections exceeding ± 80 μV, were excluded from the averaging before conducting the independent component analysis (ICA). In the ICA results, components with EOG artifacts and head movement were removed after visual inspections. Based on the topographical distribution of the grand-averaged ERP activities, the ERPs and their time windows were as follows: N2, 270−350 ms; P3, 350−460 ms. The present study selected the ERPs from site Fz for the analysis. The frontal site is representative of conflict control and does not sit directly above the premotor and motor areas of the brain, which ensures that the quality of the data is not contaminated by the button-pressing motions [68].

Two repeated-measure ANOVAs [2 (group: music training and control group) × 2 (condition: congruence and incongruence)] were conducted on the N2 and P3 amplitudes, with the group as a between-subjects factor and the condition as a within-subjects factor.

## 3. Results

There was no significant difference in the mean RAPM scores between the music training group (M = 9.92, SD = 1.22) and the control group (M = 9.84, SD = 1.29), *t*(31) = 0.21, *p* = 0.835.

### 3.1. Behavioral Results

**Figure 2:**
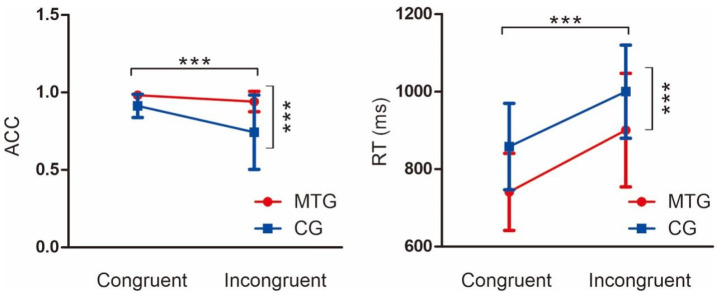
The behavioral results. MTG, music training group; CG, control group; ACC, accuracy; RT, reaction time; *** *p* < 0.001; the error bars displayed represents standard deviations.

Results on accuracy (ACC) (Figure 2) showed an interaction between the group and the condition, F (1, 49) = 6.73, *p* = 0.012, partial η^2^ = 0.12. The simple effect analysis showed that ACC in the music training group was greater than that in the control group in both conditions. The results also showed a main effect of group, F (1, 49) = 22.39, *p* < 0.001, partial η^2^ = 0.31, as ACC in the music training group was greater than that in the control group. Results also showed a main effect of condition, F (1, 49) = 17.78, *p* < 0.001, partial η^2^ = 0.27, as ACC in the congruent condition was greater than that in the incongruent condition.

The results on RT (Figure 2) showed a main effect of condition, F (1, 49) = 164.19, *p* < 0.001, partial η^2^ = 0.77, with RT in the congruent conditions being lower than those in the incongruent conditions. The results also showed a main effect of group, F (1, 49) = 11.63, *p* = 0.001, partial η^2^ = 0.19, in both conditions, as RT in the music training group was lower than that in the control group. We were unable to detect an interaction effect between group and condition, F (1, 49) = 0.57, *p* = 0.45, partial η^2^ = 0.01.

### 3.2. ERP Results

#### 3.2.1. N2

**Figure 3:**
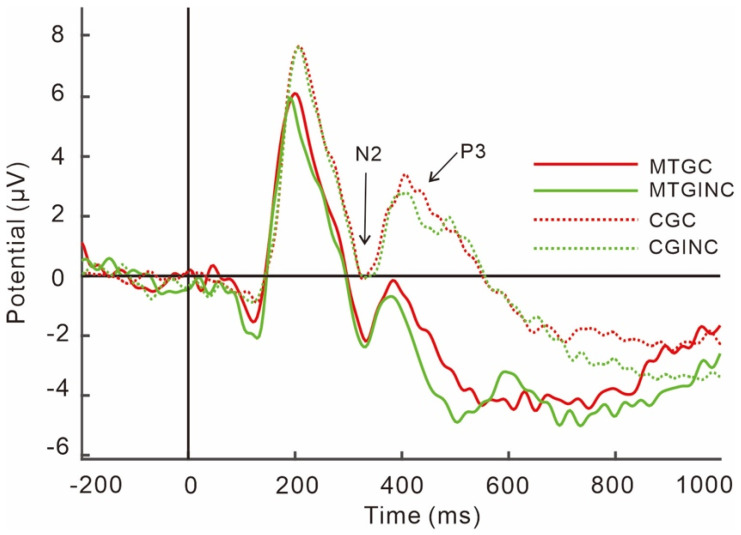
Stimuli-locked, grand average waveforms of N2 and P3 at site Fz. *Note*: MTGC, music training group in congruent condition; MTGINC, music training group in incongruent condition; CGC, control group in congruent condition; CGINC, control group in incongruent condition.

As shown in Figure 3, the results on N2 amplitudes showed a main effect of group, F (1, 49) = 4.60, *p* = 0.037, partial η^2^ = 0.09, as the N2 amplitudes in the music training group were greater than those in the control group. No interaction was found between the group and the condition, F (1, 49) = 0.005, *p* = 0.94, and no main effect of the condition was found, F (1, 49) = 1.58, *p* = 0.21, partial η^2^ = 0.03.

#### 3.2.2. P3

As shown in Figure 3, the results on P3 amplitudes showed a main effect of the condition, F (1, 49) = 4.99, *p* = 0.03, partial η^2^ = 0.09, as the P3 amplitudes in the incongruent condition were greater than those in the congruent condition. A main effect of the group was found, F (1, 49) = 11.36, *p* = 0.001, partial η^2^ = 0.19, and the P3 amplitudes in the control group were greater than those in the music training group.

## 4. Discussion

The present study examined the relationship between music training on conflict control ability in college students. Participants with music training were compared with participants who had never received formal music training, and conflict control was assessed via a Stroop task. The results found more accuracies and faster reaction times in the music training group, regardless of task condition, than the control group. Furthermore, the N2 amplitudes were higher in the music training group than in the control group. Furthermore, we discovered that in both groups, the congruent trials had P3 amplitudes larger than the incongruent trials. However, compared between the two groups, smaller amplitudes were exhibited in the control group.

Our results showed no significant difference between the music training group and the control group, which further demonstrated that when other factors are balanced, music training has beneficial effects on conflict control. Intelligence has been one of the largest confounding variables in the research field of music [69,70]. Most of the previous studies showing a significant difference in intelligence between musicians and non-musicians have adopted non-student samples, which included participants from various socioeconomics, professional, and educational backgrounds, which might have contributed to the differences in IQ [63,71]. The present study, however, did not find any differences regarding intelligence between the two groups. The discrepancy could be because the present sample consisted of students from the same university, which would imply that they are similar in age, education level, and the environment they live in. Additionally, it is common for studies investigating cognitive ability differences between musicians and non-musicians using student samples to find no significant differences in IQ test results [72,73]. The results indicate that when controlled for intelligence, there was still a difference in conflict control ability between the music training group and the control group.

The behavioral results remained consistent with previous studies [74]. In the case of the trial condition, more ACC was demonstrated in the congruent trials, which remains consistent with the near-unanimous previous behavioral results of the Stroop task, validating the enhanced task difficulty in the incongruent trials [44,58,75]. The music training group also exhibited higher ACC on the Stroop task in both the congruent and incongruent trials than the control group, indicating that the music training group outperformed the control group on the task in terms of ACC. For the RT, in both the congruent and incongruent trials, the music training group responded faster than the control group. This would provide evidence for the faster information processing speed of the music training group. Our results align with previous studies. Chen and colleagues also found that compared to non-music majors, music majors exhibited faster reactions and higher accuracy on a Stroop task [74]. Moreover, even compared with an active control group of sports training for the same length of time for 2 years, the music training group still managed to exhibit faster and more accurate responses [10]. We can conclude that incongruent trials entail greater task difficulty than congruent trials, indicated by less ACC and more RT in both groups. Notably, the music training groups’ response speed was markedly higher than the control group, in a way that their RT to incongruent trials was made at a similar speed to the control groups’ RT to the congruent trials, suggesting a pronounced enhanced processing speed, cognitive, and conflict control ability in the music training group.

The N2 and P3 potentials are proposed to be tied to inhibition and conflict mechanisms [76,77]. Past studies have concluded the validity of N2′s role as the ERP index for response conflict and conflict detection abilities, which was evidenced by the enhanced amplitudes in the incongruent trials than the congruent trials [78]. Depending on the paradigm used, the N2 recorded should be interpreted differently. For example, larger N2 in conditions where participants were instructed to inhibit a response would be most likely to indicate inhibition effort, while in conditions where participants are asked to process inconsistent information, this would indicate more attuned conflict detection [36,79]. Similarly, P3 amplitudes need to be viewed in accordance with the paradigm used too. For example, P3 amplitudes are smaller when a task demands higher cognitive functions, meaning fewer efforts are required to allocate attention to the task at hand. However, the P3 amplitudes would be enhanced if suppression of the motor response was made [80]. The present results demonstrated bigger N2 amplitudes and smaller P3 amplitudes in the music training group during a Stroop task, which remain consistent with previous findings [73,81]. More specifically, our results showed greater N2 and smaller P3 amplitudes in the music training group in both trial conditions, while exhibiting greater P3 amplitudes in the incongruent trials in both group conditions, which is identical to the results of a previous study [82].

The N2 amplitude in the music training group revealed greater negativity than the control group in both congruent and incongruent conditions. Since N2 is directly related to conflict monitoring and conflict control, the regulation of N2 means more readiness to detect novel stimuli. It has been found that music training enhances N2 amplitudes through the improvement of cognitive functions [56,83]. The larger N2 amplitudes in the music group indicate that they are more prone to detect inconsistencies in the stimuli. The greater N2 also suggests better conflict detection and control ability during the Stroop task in both congruent and incongruent situations. Combined with the behavioral results of faster responses, the results suggest that the music training exhibited faster mental processing of the Stroop task coherently over the control group.

The N3 component is associated with response inhibition and memory retrieval [84,85]. The current paradigm used only access for its inhibition ability. A larger P3 amplitude in the control group would supposedly provide evidence for their ability to withhold a response. However, the paradigm was designed to ask participants to elicit a correct response instead of suppressing one. The larger P3 would indicate that the control group needed more cognitive effort to do so. This is also consistent with the current results that incongruent trials elicited greater P3, meaning the harder the perceived task difficulty, the larger the P3 amplitudes. The smaller P3 amplitude in the music training group in both congruent and incongruent conditions indicated that the task did not require the same amount of cognitive energy as it did in the control group, which stays consistent with previous findings [86,87].

Ultimately, the findings showed a link between music training and improved conflict control capacity and processing speed. Based on the ERP results, we now have a clearer picture of how they helped the music training group outperform the control group. The N2 amplitude of the music training group was bigger than that of the training group, which the smaller P3 also reflects. Based on the results, we make the following reasoning: When practicing music, musicians need to pay attention to the coordination between hands and eyes at the same time and be attentive to the strength and position of the body while staying on track with the music, exercising the conflict control ability in the usual practice.

Some limitations of the study need to be acknowledged. Firstly, the study lacks an active control group. The results of the study do not provide inductive evidence for other forms of extracurricular training, and we remain unclear about the differences between the effects of various training. Future studies could engage various musical, athletic, and or artistic training to provide a more comprehensive understanding of the effects. The study had another limitation—it did not account for different variables that could have impacted the impact of music training on conflict control, such as years spent in musical studies and musical skills.

It has also been shown that individuals who choose to pursue a music degree are associated with various factors, including openness to new experiences, demographics, and conscientiousness, which would influence cognitive abilities [2,61,88,89]. Future studies could investigate those potential variables and examine how they may or may not have mediated the relationship between music training and conflict control. Lastly, the study did not enroll an adequate sample size to test for the effects of different instruments on conflict control. Since different instruments pose different levels of cognitive demands to the player, future research could be carried out to involve a larger sample size to examine how the factor of instruments influences the relationship.

## 5. Conclusions

The results suggested that incorporating and adopting music training can have an advantageous influence on an individual’s development by helping his/her conflict control capabilities. Individuals with music training backgrounds outperformed the control group in the Stroop task in terms of accuracy and reaction time. Moreover, compared to controls, the music training group also exhibited greater N2 amplitudes, which indicated better conflict detection and monitoring abilities. They also exhibited smaller P3 amplitudes, which indicated less cognitive effort when required to selectively pay attention to the relevant stimulus. Overall, our findings support our hypothesis that there is an advantageous relationship between music training and conflict control ability. The study provided further explanation into the research field of music training and executive functions. The findings aid real-world implications of music training programs and music education by demonstrating their positive outcomes on on an individual’s cognitive ability.

## Data Availability

The data presented in the study are available upon reasonable request from the corresponding authors.
